# Detection of lung lesions in breath-hold VIBE and free-breathing Spiral VIBE MRI compared to CT

**DOI:** 10.1186/s13244-021-01124-0

**Published:** 2021-11-24

**Authors:** Susann-Cathrin Olthof, Christian Reinert, Konstantin Nikolaou, Christina Pfannenberg, Sergios Gatidis, Thomas Benkert, Thomas Küstner, Patrick Krumm

**Affiliations:** 1 Department of Diagnostic and Interventional Radiology, University Hospital of Tuebingen, Hoppe-Seyler-Straβe 3, 72076 Tuebingen, Germany; 2 MR Applications Predevelopment, Siemens Healthcare GmbH, Allee am Roethelheimpark 2, 91052 Erlangen, Germany

**Keywords:** Spiral VIBE, Ultrashort echo time, MR lung nodule detection, Tomography (X-ray computed)

## Abstract

**Background:**

Detection of pulmonary nodules in MRI requires fast imaging strategies without respiratory motion impairment, such as single-breath-hold Cartesian VIBE. As patients with pulmonary diseases have limited breath-hold capacities, this study investigates the clinical feasibility of non-Cartesian Spiral VIBE under free-breathing compared to CT as the gold standard.

**Methods:**

Prospective analysis of 27 oncological patients examined in PET/CT and PET/MR. A novel motion-robust 3D ultrashort-echo-time (UTE) MR sequence was evaluated in comparison with CT and conventional breath-hold MR. CT scans were performed under breath-hold in end-expiratory and end-inspiratory position (CT ex, CT in). MR data was acquired with non-contrast-enhanced breath-hold Cartesian VIBE followed by a free-breathing 3D UTE Spiral VIBE. Impact of respiratory motion on pulmonary evaluation was investigated by two readers in Cartesian VIBE, followed by UTE Spiral VIBE and CT ex and the reference standard of CT in. Diagnostic accuracy was calculated, and visual image quality assessed.

**Results:**

Higher detection rate and sensitivity of pulmonary nodules in free-breathing UTE Spiral VIBE in comparison with breath-hold Cartesian VIBE were found for lesions > 10 mm (UTE Spiral VIBE/VIBE/CT ex): 93%/54%/100%; Lesions 5–10 mm: 67%/25%/ 92%; Lesions < 5 mm: 11%/11%/78%. Lobe-based analysis revealed sensitivities and specificities of 64%/96%/41% and 96%/93%/100% for UTE Spiral VIBE/VIBE/CT ex.

**Conclusion:**

Free-breathing UTE Spiral VIBE indicates higher sensitivity for detection of pulmonary nodules than breath-hold Cartesian VIBE and is a promising but time-consuming approach. However, sensitivity and specificity of inspiratory CT remain superior in comparison and should be preferred for detection of pulmonary lesions.

## Key points

- Clinical feasibility of a free-breathing 3D UTE Spiral VIBE sequence.
- Superior detection rate for lesions > 10 mm in Spiral VIBE compared to VIBE.
- Inferior detection rate of Spiral VIBE compared to CT as gold-standard.

## Introduction

Magnetic resonance imaging (MRI) of the air-filled lungs suffers from motion artifacts due to the cyclic heartbeat and respiration [1]. The low proton density of only approximately 800 g for both lungs and fast T2* decay of air result in a low lung signal intensity [2] reflected in a low signal-to-noise ratio (SNR) in the resulting image. Furthermore, susceptibility artifacts caused by the interface of different tissue types lead to field inhomogeneities with short T2 and T2* lung signal in the chest [2].

To overcome the two major problems of low SNR and impact of respiratory motion, previous works aimed at providing higher SNR with the ultrashort echo time (UTE) technique for 3D stack-of-radials or 3D radial imaging [3–7] with reported sensitivities of 73–93% for lung nodule detection in comparison with the gold standard of radiation-dependent computed tomography (CT) imaging [3]. Moreover, echo time reduction was also achieved with central pointwise encoding and stack-of-radials acquisition (PETRA) to evaluate pulmonary pathologies such as nodules, cystic fibrosis and pulmonary embolisms [8]. Respiratory motion was resolved by freezing respiratory motion via fast single [9] or multiple [10] breath-hold acquisitions.

Even imaging under free-breathing was performed using a fast and motion-robust UTE T1-weighted 3D gradient echo sequence with non-Cartesian stack-of-spirals trajectory readout including short echo times to capture fast T2* decay (UTE Spiral VIBE) [11].

The aim of this study was to investigate the diagnostic accuracy for detection of pulmonary nodules with UTE Spiral VIBE sequence acquired under free-breathing conditions in clinical routine without i.v. contrast application. We hypothesized a prolonged scan time of UTE Spiral VIBE may be justified for better lung lesion detectability in comparison with: 1) end-expiratory breath-hold Cartesian VIBE acquired on MRI and baseline reference, 2) CT in inspiration and 3) expiration.

## Methods

### Study cohort

Prospective, consecutive analysis of 27 clinically induced PET/CT examinations with following, voluntary PET/MR examination was performed, independent of the pulmonary findings in chest CT. Patients gave written consent, and the local review board approved this prospective study. PET/MR acquisition included VIBE and UTE Spiral VIBE sequences for lung evaluation. Mean patient age was 65.6 ± 10 years (range 45–83 years), 7 females (26%). Primary diagnosis for 24 ^18^F-FDG PET/CT examinations were lung lesions (*n* = 16), non-Hodgkin lymphoma (*n* = 3), cholangio-cellular carcinoma (*n* = 1), breast-cancer (*n* = 1), melanoma (*n* = 2) and rectum cancer (*n* = 1), as well as neuroendocrine tumors NET (*n* = 2, ^68^ Ga-DOMITATE PET), prostate cancer (*n* = 1 ^68^ Ga-PSMA PET). Exclusion criteria were aged under 18 years and contraindications for MR imaging and pleural effusion.

### Data acquisition

**Fig. 1:**
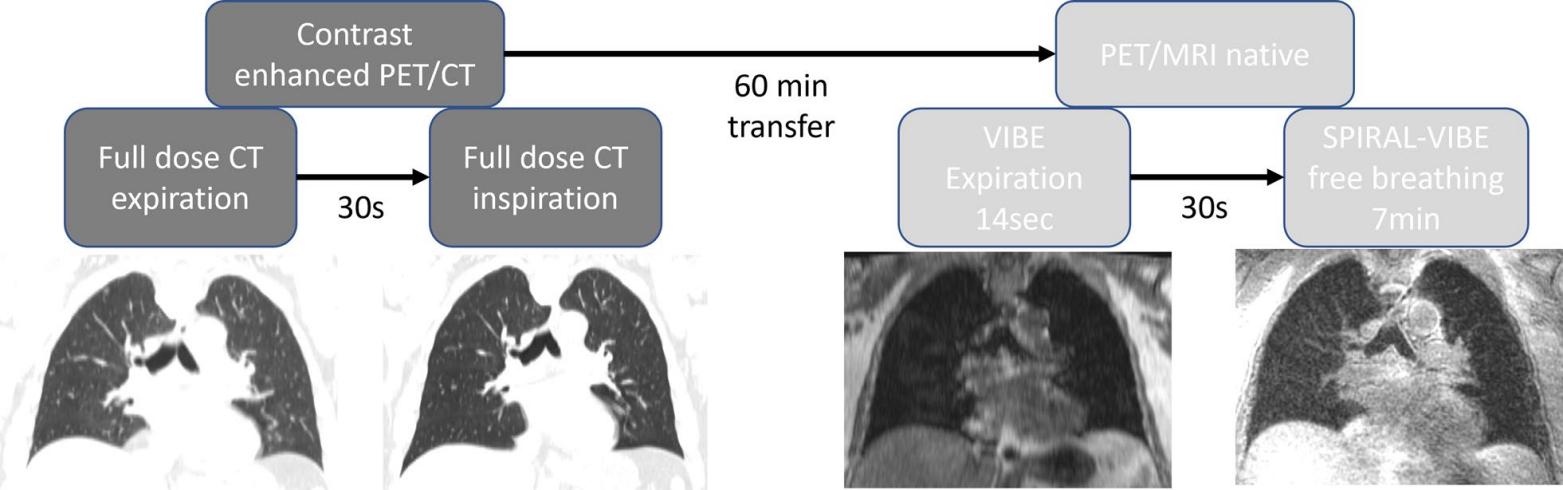
Flowchart of examination sequence in integrated PET/CT and PET/MRI scanners

All images were acquired on a 3 T PET/MR scanner in supine position with both arms besides the body (Biograph mMR, Siemens Healthcare, Erlangen, Germany) and on a PET/CT scanner with elevated arms on the same day, applying standardized examination and acquisitions parameters (Biograph mCT, Siemens Healthcare, Knoxville, TN, USA). Details of the examination protocol and scan parameters are depicted in Table 1 and Fig. 1.

**Table 1:** Overview of the MR and CT data acquisition details

	VIBE	UTE Spiral VIBE		CT expiration	CT inspiration
Resolution [mm^3^]	1.3 × 1.3 × 3	1.5 × 1.5 × 1.5 (isotropic resolution)	Resolution [mm^3^]	0.8 × 0.8 × 0.7	0.8 × 0.8 × 0.7
Matrix	320 × 240	320 × 320	Matrix	512 × 512	512 × 512
Repetition time [ms]	3.9	3.2	Peak tube voltage [kV]	120	120
Echo time [ms]	2.5	0.05	Tube current [mAs]	Dose modulated	Dose modulated
Acquisition time [s]	14 ± 1	409 ± 15	Convolution kernel	I31f/2	I30f/2
Bandwidth [Hz/pixel]	1185	975			
Flip angle [°]	10	5

#### MRI

3D T1-weighted spoiled gradient echo sequence (VIBE) was acquired in expiration phase in axial plane, with Cartesian trajectory, centric reordering, without parallel imaging. Two echoes were recorded for DIXON-based fat–water separation. Field-of-view placement and orientation were similar to CT. Image acquisition time was 14 s for breath-hold VIBE. Additionally, a prototypical UTE Spiral VIBE acquisition was performed without i.v. contrast application (as patients already received CT contrast media prior on the same day), using spiral sampling in the kx-ky plane and Cartesian sampling in kz direction. By employing short rectangular pulses, a center-out acquisition, and variable TE encoding [12], an echo time of 50 us could be achieved. Therefore, compared with Cartesian VIBE, faster relaxing T2* tissues can be captured. To reduce the number of through-plane phase-encoding steps, images were acquired in coronal orientation. Data acquisition was performed during free-breathing. To avoid corresponding motion artifacts, prospective respiratory gating was performed, for which a self-navigator signal based on intermitted navigator pulses in the SI direction was evaluated during scanning, and scanning was automatically stopped once sufficient data had been acquired in end-expiratory state. Acquisition time therefore depends on the patient’s breathing pattern and ranged from 6:30 to7 minutes per patient depending on the acquired slabs.

#### CT

For reference standard of lung lesion detection and evaluation, chest CT in inspiration (CT in) was used, which is part of our house-intern standard PET/CT protocol. Additional whole-body CT in expiration (CT ex), clinically necessary for the PET attenuation correction, served as comparative CT scan to the gold standard. Both scans were acquired after i.v. contrast agent application (120 ml, flow rate 2.5 ml/s; Ultravist 370 Bayer Healthcare Pharmaceuticals Berlin, Germany).

### Image evaluation

All data were analyzed in axial plane starting with VIBE, UTE Spiral VIBE, CT in expiration and inspiration using syngo.via Client 5.1 for PET/MRI and PET/CT examinations by two blinded readers with 7 and 9 years of oncological experience including fellowships in nuclear medicine (syngo.MM Oncology VB30A, Siemens Healthcare, Erlangen, Germany; Fig. 1). Calcified lesions and dystelectases were omitted. The longest lesion diameter was measured in lung window settings for CT. If the lesion was not found in inspiratory chest CT, the lesion was defined as ‘false positive,’ whereas ‘false negative’ lesions were not detected in any method except inspiratory CT. Pulmonary lobes without any lesions in inspiratory chest CT were counted as ‘true negative.’ Additionally, image quality was assessed for both CT and MRI imaging applying a four-point scale according to Kumar et al. [13]. Evaluation criteria included the sharpness, potential artifacts, image noise and overall image quality with scores of (1) for perfect image quality, (2) for clinically sufficient image quality, (3) for moderate image quality and (4) for impaired image quality. Lung lesions were analyzed according to their number, their lobe location and their largest size and categorized in smaller than 5 mm, 5 to 10 mm and over 10 mm.

### Statistical analysis

Categorical, continuous variables and frequencies are given as means with standard deviation (SD). Sensitivity (Sn) and specificity (Sp) were calculated for VIBE, UTE Spiral VIBE and CT ex in comparison with the gold standard of CT in for the whole lung in each patient (lesion-based analysis) and according to the lobe (lobe-based analysis). Intraclass correlation coefficients were created for inter-reader agreement for lesion detection in Spiral VIBE. For objective (Spiral) VIBE sequence evaluation, a coefficient of variation (mean and SD) was calculated via SD of the index lesion divided through the mean of the index lesion per patient. Furthermore, the contrast difference ratio (mean and SD) was calculated via contrast-to-noise ratio (contrast between index lesion and lung surrounding) divided through the signal-to-noise ratio for each index lesion per patient. All statistical analyses were performed with MedCalc (Version 12.6 MedCalc Software, Ostend, Belgium).

## Results

In 27 patients, 99 lung lesions were detected in total in the inspiration chest CT, which served as gold standard. Lung lesions per patient varied from one to nine, with a mean value of 3.4 per patient (SD 2.6). Size varied from 2 to 122 mm (mean 13 mm, SD 22 mm).

**Fig. 2:**
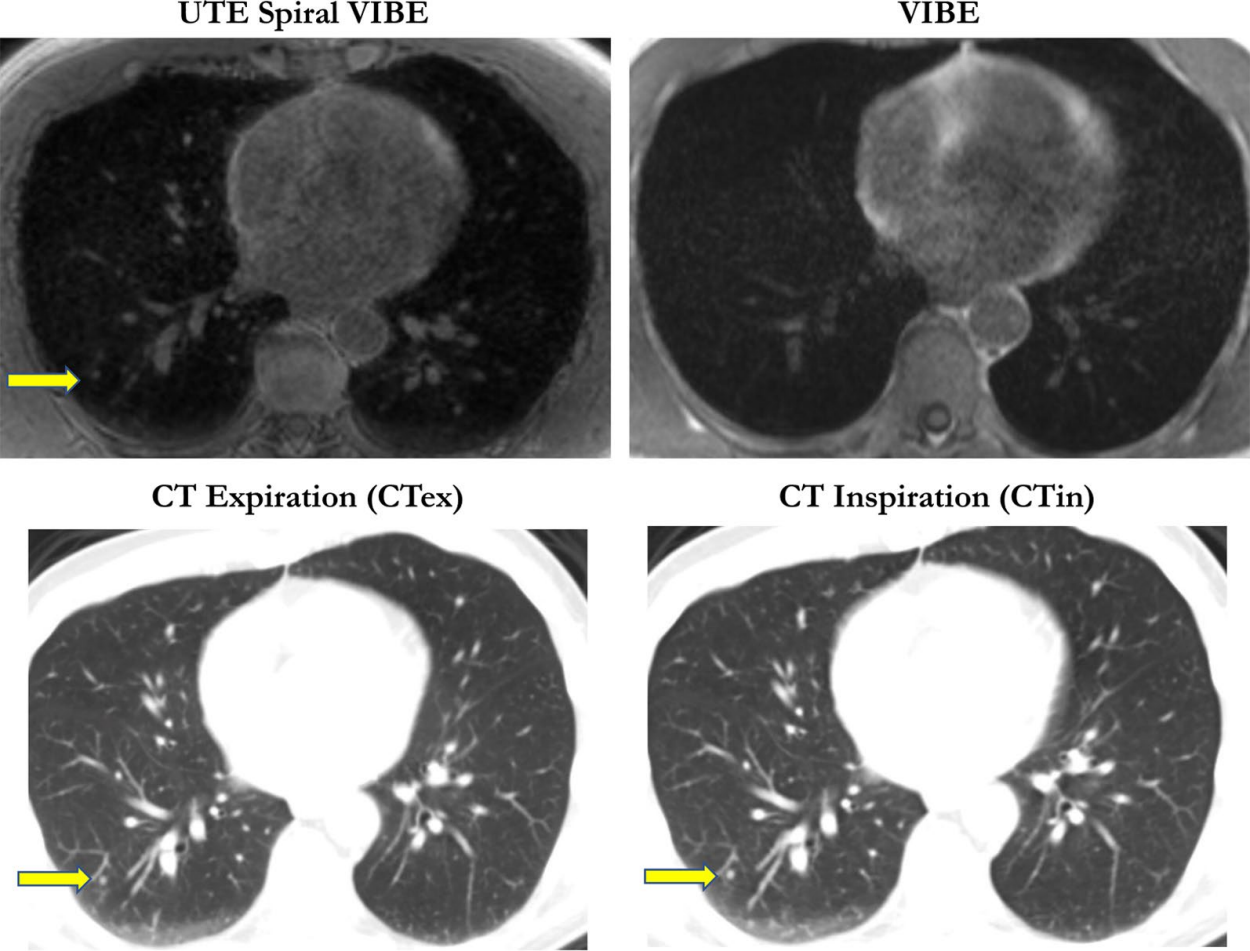
Pulmonary nodule in the right lower lobe was detected in UTE Spiral VIBE and both CT examinations (arrow; 3 mm). However, the lesion was not visible in VIBE in a patient with rectal carcinoma

**Fig. 3:**
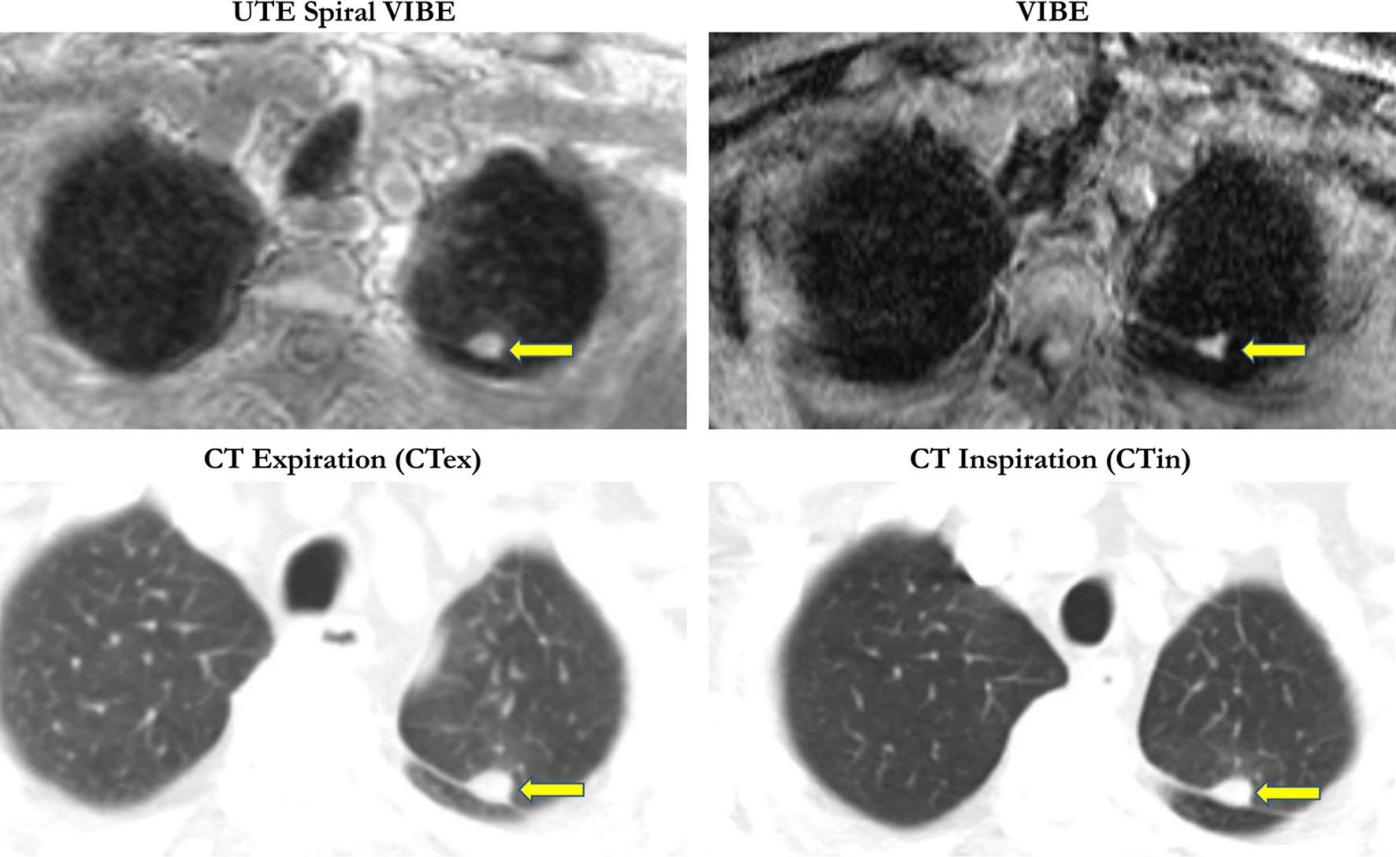
Perifissural bronchial carcinoma in the upper lobe was detected in UTE Spiral VIBE, VIBE and CT imaging; (arrow; 15 mm)

Representative cases of good and difficult pulmonary node detection are given in Figs. 2 and 3.

### Lesion-based analysis

CT in expiration revealed the highest detection rate for pulmonary lesions in comparison with the reference standard of CT in inspiration (88%). Accordingly, UTE Spiral VIBE was superior to VIBE for pulmonary lesion detection (47% vs. 26%), especially for the detection of lesions between 5 and 10 mm (67% vs. 25%) and lesions over 10 mm (93% vs. 54%). However, no difference was stated between both MR imaging techniques for smaller nodules of 5 mm (11%; Table 2).

**Table 2:** Overview of the detected lesions according to MRI sequence VIBE and Spiral VIBE as well as CT in expiration in comparison with the gold standard of CT in inspiration both lesion- and lobe-based

	**VIBE**	**UTE Spiral VIBE**	**CT expiration**
**Detection rate of all lesions**	26/99	47/99	87/99
Sensitivity	26%	47%	88%
(95% CI)	(17–38)	(32–59)	(70–107)
**Detection rate according to lesion size**			
< 5 mm	5/47	5/47	37/47
	11%	11%	79%
5–10 mm	6/24	16/24	22/24
	25%	67%	92%
> 10 mm	15/28	26/28	28/28
	54%	93%	100%
**Lobe-based analysis**	24/58	37/58	54/58
Sensitivity	41%	64%	93%
(95% CI)	(41–70)	(26 – 51)	(41–70)
**Lobe-based analysis**	74/77	74/77	77/77
Specificity	96%	96%	100%
(95% CI)	(58–93)	(58–94)	(61–96)

### Lobe-based analysis

Analysis of the detected lesions per lobe revealed higher detection rate for VIBE (41%) and UTE Spiral VIBE (64%) as well as CT in expiration (93%) in comparison with the gold standard of chest CT in inspiration. Specificity of both VIBE and Spiral VIBE was 96% and for chest CT in expiration 100% (Table 2).

CT in expiration showed the highest sensitivity for lung nodule detection (98%) in comparison with the gold standard, independent of the lesion localization. Both MR sequences were impaired for lesions next to the fissura horizontalis and obliqua. However, UTE Spiral VIBE was superior to VIBE with a sensitivity of 46% vs. 27% (Table 3).

**Table 3:** Synopsis of the detected lung lesions according to the anatomical distribution

Detection rate according to localization	VIBE	UTE Spiral VIBE	CT expiration
Right upper lobe	9/24	11/24	21/24
Middle lobe	0/8	1/8	8/8
Right inferior lobe	5/21	6/21	17/21
Right hilum	2/2	2/2	2/2
Left upper lobe	3/19	13/19	17/19
Left inferior lobe	6/17	13/17	15/17
Left hilum	1/1	1/1	1/1
Fissura horizontalis/obliqua	0/7	0/7	6/7
Central localization	23/60	38/60	70/60
Subpleural localization	3/39	9/39	17/39

### Image quality assessment

Image quality was assessed for each imaging modality with a score from 1 for perfect image quality up to a score of 4 for impaired image quality including the evaluation of the overall image quality. The highest image quality was observed in the gold standard of CT in inspiration. Although UTE Spiral VIBE showed lowest numbers for score 1 image quality in general, the clinical applicable image quality of scores 1 and 2 were higher compared to VIBE (8.5% + 70.2% = 78.7% vs. 23% + 27% = 50%; Table 4). Mean coefficient of variation was, respectively, 0.170 ± 0.082 and 0.112 ± 0.045, respectively, for VIBE and Spiral VIBE. Mean contrast difference ratio was 0.906 ± 0.028 and 0.848 ± 0.061 for VIBE and Spiral VIBE.

**Table 4:** Overview of the image quality for the MRI and CT examination modalities

Score	VIBE	UTE Spiral VIBE	CT expiration	CT inspiration
1	6/26 (23%)	4/47 (9%)	19/87 (22%)	80/99 (81%)
2	7/26 (27%)	33/47 (70%)	62/87 (71%)	17/99 (17%)
3	13/26 (50%)	10/47 (21%)	6/87 (7%)	2/99 (2%)
4	0	0	0	0

### Inter-reader agreement

The absolute inter-reader agreement for nodule detection was 0.818 (95% CI 0.7499–0.8697).

## Discussion

This prospective study demonstrates superiority of free-breathing UTE Spiral VIBE in comparison with breath-hold VIBE for lung nodule detection in a clinical routine setting at 3 T. For lesions over 10 mm, UTE Spiral VIBE reveals highest detection rates for lung nodules in MRI, comparable to chest CT in expiration. As a hallmark of this study, both MRI and CT imaging examinations were performed in expiration, resulting in a directly comparable image evaluation without any distortions due to different imaging settings.

Sensitivities for lung nodule detection at 3 T are with 27% for VIBE and 48% for UTE Spiral VIBE in this first readers’ experience study inferior to literature data (55% [14]). This might be caused by the high rate of small lung lesions under 5 mm in our study in the gold standard of CT imaging (47/99, 48%). In accordance, current clinical studies [9] and a current animal model indicate MRI suitable for detection of lung lesions larger than 4 mm [15].

Furthermore, the difference in examination protocols, like the slice thickness and the MR field strength, is crucial. Chassagnon reported significantly lower SNR and contrast-to-noise ratios (CNR) at 1.5 T in comparison with 3 T in ten volunteers [16]. Higher SNR is also obtained after i.v. contrast media application [17], explaining the difference in pulmonary detection rates in the literature. Other reasons might be the examination setting, as our patients underwent non-enhanced MR examination directly after PET/CT scan on the same day, in comparison with Ohno, where patients received both examinations within one week [7]. Here, comparable detection rates between CT with standard and reduced dose as well as UTE were described in 52 patients with similar lung detection diameters to ours (mean 13 vs. 12 mm; SD 22 vs 7.3 mm [7]). However, the reported high sensitivities of lung nodule detection of 73% in 82 pulmonary nodules in eight patients examined with UTE [18] are less meaningful without the reported specificities.

With regard to image quality, UTE Spiral VIBE in free-breathing acquisition seems to be more robust for clinical application in comparison with breath-hold VIBE, as the total amount of score-1 and score-2 image qualities was higher (78% vs. 49%). Improved coefficient of variation with similar contrast ratios for UTE Spiral VIBE in comparison with VIBE supports the observation of a reduced noise floor. Accordingly, UTE Spiral VIBE is less prone to motion artifacts with reported comparable morphological information to CT [19]. Thus, the longer acquisition time of approximately 7 min for UTE Spiral VIBE in comparison with 14 s for VIBE seems effective, especially in the whole-body PET/MRI staging for oncological patients with a reduced general and in some cases pulmonary condition. Further potential application for Spiral VIBE might lie in the therapeutic field of radiotherapy planning of the primary lung tumor, if clearly visible, thereby reducing unnecessary breathing stops and further radiation exposure for the patient. However, the main potential role for MR lung examination lies especially in the field of pediatric imaging, to replace low-dose chest CT for lung evaluation. Here, the free-breathing approach can be beneficial especially for age groups that cannot follow breath-hold commands.

Since low-dose and ultra-low-dose CT protocols in inspiration with iterative reconstruction algorithms offer very high sensitivities above 90% and radiation dose of 0.1 mSv, the CT approach also seems a suitable pathway without relevant radiation exposure [20, 21]. The question arises whether it may be more helpful to further improve lung evaluation with low-dose protocols in CT as the diagnostic gold standard, for any MRI approach will have inherent drawbacks due to susceptibility with the organ of interest full of air. A reasonable approach for staging MRI may include a lung-specific sequence with the duration of one breath-hold, but it can currently not replace the high sensitivity of a lung CT.

We acknowledge several limitations of this study. The patients’ positioning of the arms beneath the body for MR sequences potentially affects the imaging quality of the lung, especially when compared to PET/CT with elevated arms. However, this is a methodological issue, which affects any other comparable study. Furthermore, VIBE sequence was acquired in axial, UTE Spiral VIBE in coronal orientation, impeding the sensitivities for lung nodule detection in VIBE as the respiration occurs in the *z*-axis [10]. Our study was focused on an image-guided detection of lung lesions, without histopathological correlation of the analyzed MR lesions, regarding the clinical relevance. Furthermore, lesions were not analyzed for the morphology (ground glass nodules, part-solid nodules, and solid nodules). This could be crucial for risk assessment of pulmonary nodules in the future.

## Conclusion

Free-breathing UTE Spiral VIBE indicates higher sensitivity for detection of pulmonary nodules than breath-hold VIBE and is a promising but time-consuming approach. However, sensitivity and specificity of inspiratory CT remains superior in comparison and should be preferred for detection of pulmonary lesions in adults. However, we see a potential role for UTE Spiral VIBE for imaging in dose-restricted cohorts, e.g., pediatric patients.

## Data Availability

The datasets used during the current study are available from the corresponding author on reasonable request.

## Abbreviations

^18^F-FDG PET/CT: ^18^Fluorine fluorodeoxyglucose positron-emission tomography/computed tomography
CAIPIRINHA: Controlled aliasing in parallel imaging results in higher acceleration
CNR: Contrast-to-noise ratio
CT: Computed tomography
EX: Expiration
FOV: Field of view
GRE sequence: Gradient echo sequence
INS: Inspiration
MRI: Magnetic resonance imaging
NET: Neuroendocrine tumor
PETRA: Pointwise encoding and stack-of-radial acquisition
PSMA: Prostate-specific antigen
SE sequence: Spin echo sequence
SNR: Signal-to-noise ratio
T: Tesla
TE: Echo time
TR: Repetition time
UTE: Ultrashort echo time
VIBE: Volumetric interpolated breath-hold examination
